# Supplementary material for: Improving predictions of convective storm wind gusts through statistical post-processing of neural weather models
Source: NPJ Nat Hazards. 2025 Nov 7;2(1):100. doi: 10.1038/s44304-025-00142-y (PMC12594611; doi:10.1038/s44304-025-00142-y)
Supplement: Supplementary file 1 — Supplementary Information [file 44304_2025_142_MOESM1_ESM.pdf]

Supplementary information
Improving Predictions of Convective Storm Wind Gusts through Statistical Post-Processing of Neural Weather Models

S1: Oscillations in model performance

At first glance, the CRPSS in Fig. 2b oscillates as a function of lead time. Here we clarify what we mean by "oscillations", examine them year by year, and explain why they arise from the Pangu-Weather architecture and, to a lesser extent, from choices in the post-processing models. Although sampling effects might be suspected, Supplementary Fig. 1 shows that the same patterns recur in every year, which argues against sampling as the primary cause.

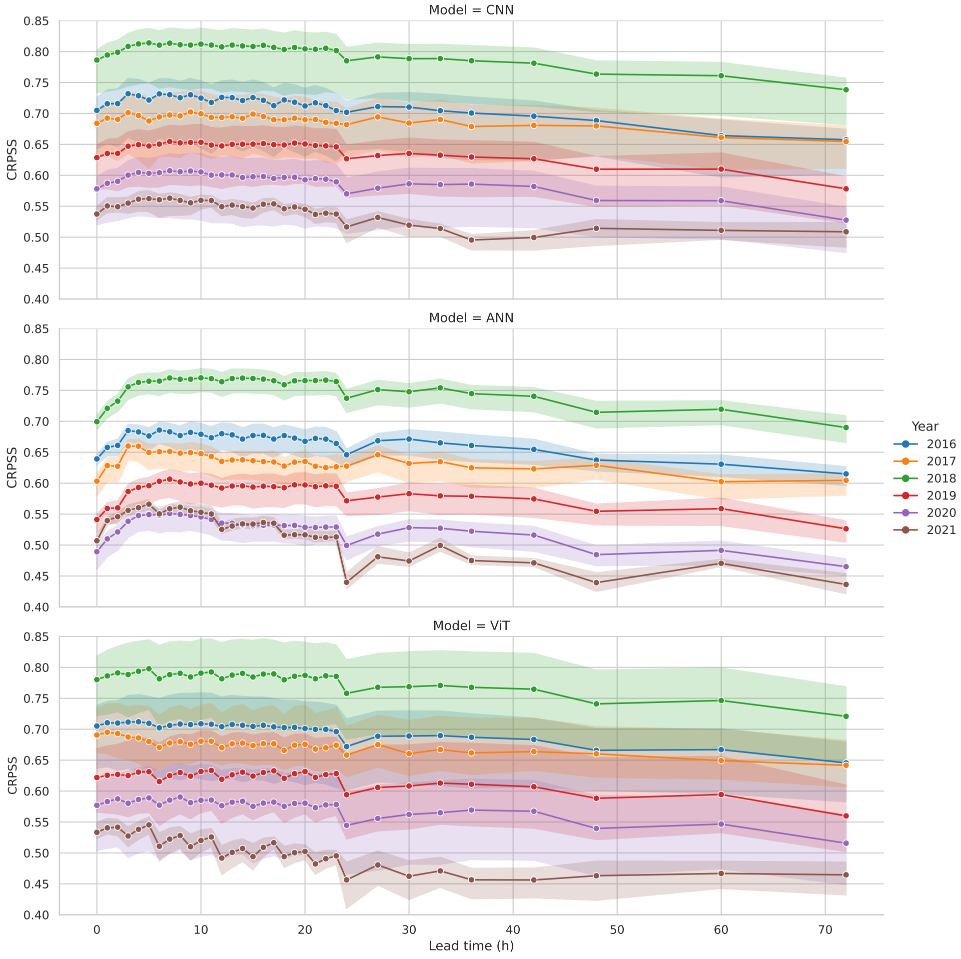

Supplementary Figure 1: CRPSS by lead time for the CNN, ANN, and ViT, shown separately for 2016–2021. Two robust features appear across all years and methods: (i) a sawtooth pattern for lead times < 24 h, caused by different short-range step compositions in Pangu-Weather; and (ii) a drop at 24 h, caused by the switch to the 24 h submodel.

Two distinct features are present: (i) a sawtooth behaviour for lead times below 24 h, and (ii) a discrete drop in skill at exactly 24 h. Both appear across years (2016–2021) and across post-processing methods (CNN, ANN, ViT).

First, Pangu-Weather uses different submodels at different horizons and composes them to reach a target lead time with a minimal number of steps [1], with oscillations also reported by [2]. Adjacent lead times can therefore be produced by different step compositions (e.g., 6+1 h vs. 3+3+1 h), which changes error characteristics slightly from one lead time to the next. This architectural choice explains the sawtooth pattern below 24 h.

Second, at 24 h the system switches to the dedicated 24 h submodel. That switch introduces a change in error properties and coincides with a notable decrease in skill that is visible in all years and methods. Because these two features are aligned with the Pangu submodel boundaries and persist across years with different storm samples, they are best attributed to the driving neural weather model rather than to sampling variability.

Post-processing models largely transmit these Pangu biases but can modulate their amplitude. In the CNN and ANN, L2 regularisation and an explicit temporal block keep temporal encodings in our post-processing models influential, which tends to smooth the sawtooth. In contrast, the ViT (dropout in the spatial block, no L2) relies more heavily on spatial features and mirrors Pangu's lead-time structure more closely. The VGAM, trained independently at each lead time, reproduces the Pangu/ViT pattern most directly because it does not share information across lead times.

In summary, the pre-24 h sawtooth arises from different short-range step compositions in Pangu-Weather, while the 24 h jump reflects the switch to the 24 h submodel. Post-processing does not create these patterns; it mainly inherits them, with minor smoothing when temporal conditioning and regularisation are stronger.

S2: CRPSS stabilisation for longer lead times

Figure 2b also shows a stabilisation of the CRPSS at longer lead times, while the CRPS increases with lead time. This behaviour arises because CRPSS is a normalised skill score relative to climatology: when both the forecast CRPS and the climatological CRPS degrade at similar rates, the relative improvement can plateau even as absolute errors grow. In our case, the plateau reflects persistent medium-range skill in the Pangu-Weather driver, particularly for 10 m wind fields that strongly inform our wind gust predictions.

This interpretation is supported by a comparison to direct forecasts based only on ERA5-derived initial conditions. If the CRPSS plateau were independent of the driver, similar stabilisation would appear for the direct baselines. Instead, as Supplementary Figure 2 shows (and as in Figure 2c of the main text), at long lead times the CRPSS of direct forecasts that do not post-process Pangu-Weather tends towards zero. By contrast, Pangu-based post-processing maintains positive CRPSS through 48–72 h.

Due to computational constraints, we did not train beyond 72 h. At longer horizons we expect CRPSS to decline as the driver's predictability decreases, consistent with the trend observed for our direct forecasting baselines.

S3: Performance for severe gusts: Sensitivity to filtering procedure

Post-processing model performance across wind gust speeds was computed for different filters. Supplementary Figures 3, 4 and 5 illustrate that most models yield positive CRPSS across gust values, which means they beat climatology even in extreme situations. Thresholds are always based on the following quantile levels: 0.5, 0.75, 0.9, 0.95, 0.99, 0.999.

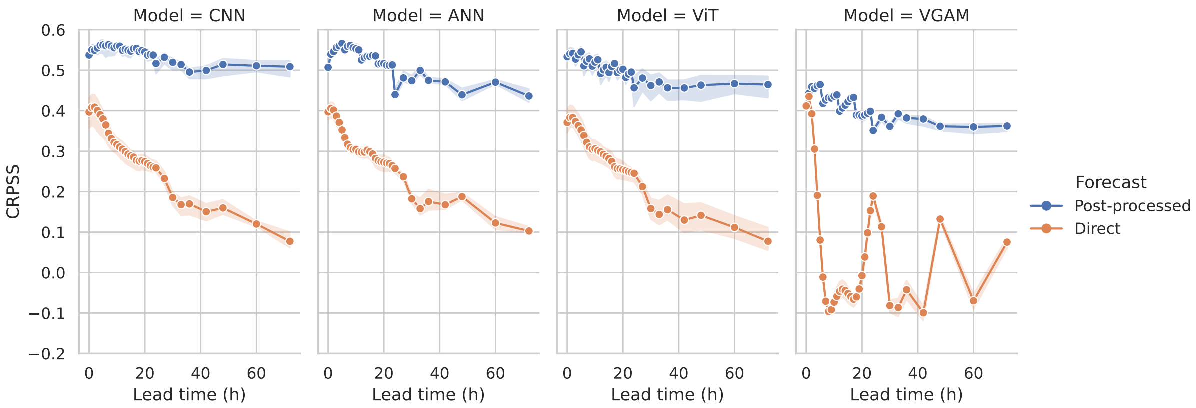

Supplementary Figure 2: CRPSS by lead time for each model (VGAM, ANN, ViT, CNN): direct forecasts from ERA5-derived initial conditions versus post-processing of Pangu-Weather. For VGAM, the curve dips below zero at some lead times. Post-processed Pangu-Weather retains positive skill and tends to plateau by 48–72 h, whereas direct baselines approach CRPSS = 0.

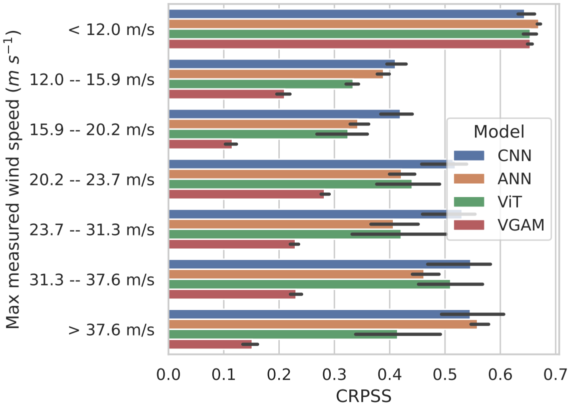

Supplementary Figure 3: CRPSS for filtered wind gusts based on the maximum wind gust observed in the cluster at each time step.

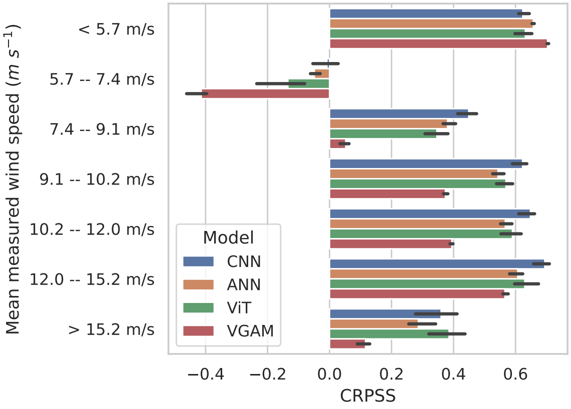

Supplementary Figure 4: CRPSS for filtered wind gusts based on the mean wind gust observed in the cluster at each time step.

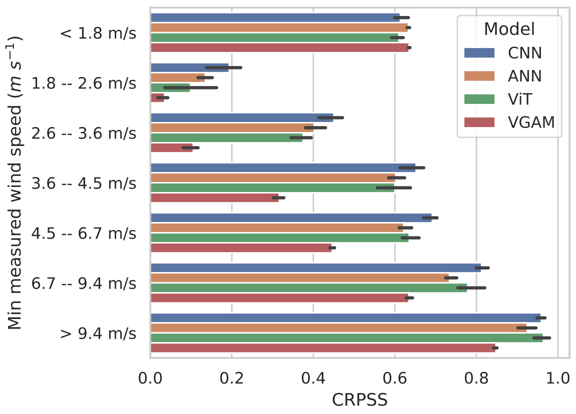

Supplementary Figure 5: CRPSS for filtered wind gusts based on the minimum wind gust observed in the cluster at each time step.

S4: Performance across regional clusters

Relative to local climatology, Supplementary Figure 6 shows that the post-processing models (except VGAM) achieve higher CRPSS over the Alps and southern plains than over the northern and western plains. This suggests that climatology is more dispersed over the Alps and southern plains—where extremes are more frequent—than north of the Alps. However, Supplementary Figure 7 shows that in absolute terms (CRPS; lower is better), models perform better in northern Switzerland, where conditions are more predictable.

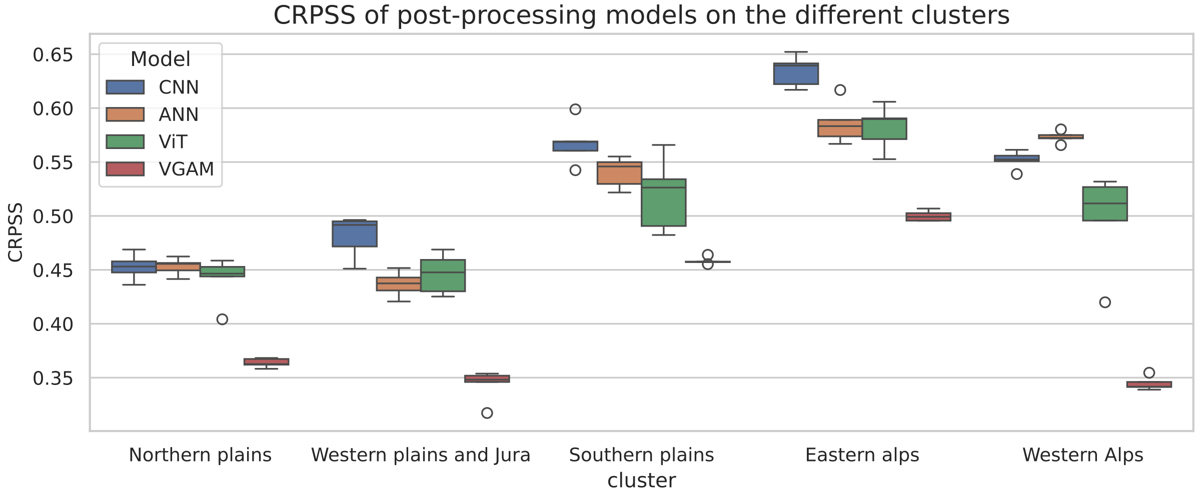

Supplementary Figure 6: CRPSS by region. Post-processing improves upon local climatology more over the Alps and southern plains.

S5: Discussion: Choice of the test year

Although only one test year might raise questions regarding the limited size of the test set, several reasons led to this choice that we believe is reasonable.

First, Figure 8 illustrates that the distribution of convective storm days in 2021 is comparable to those in the 2016–2020 training period, indicating that it is climatologically representative in terms of storm frequency.

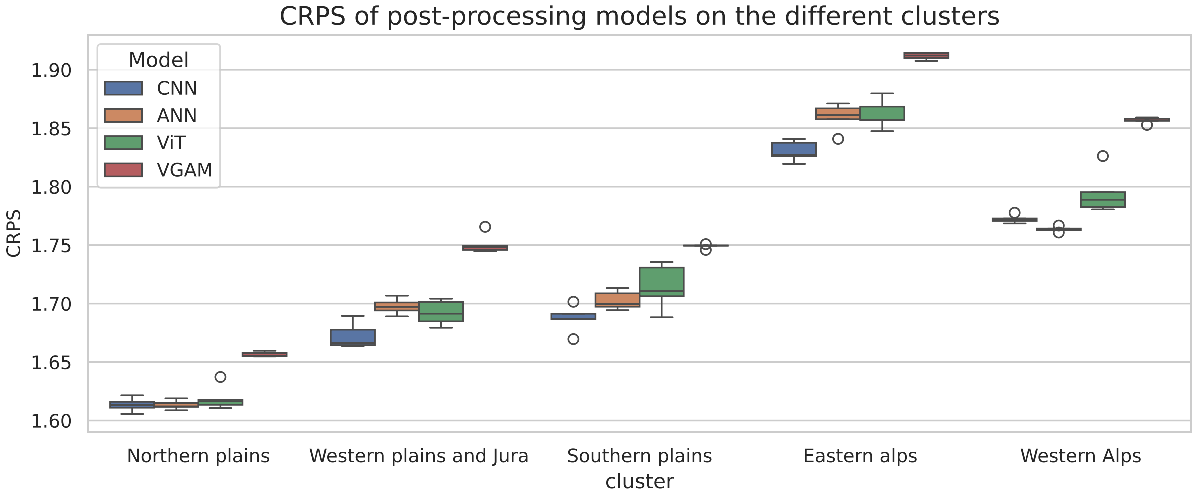

Supplementary Figure 7: CRPS by region (lower is better). Errors are larger over the Alps and southern plains.

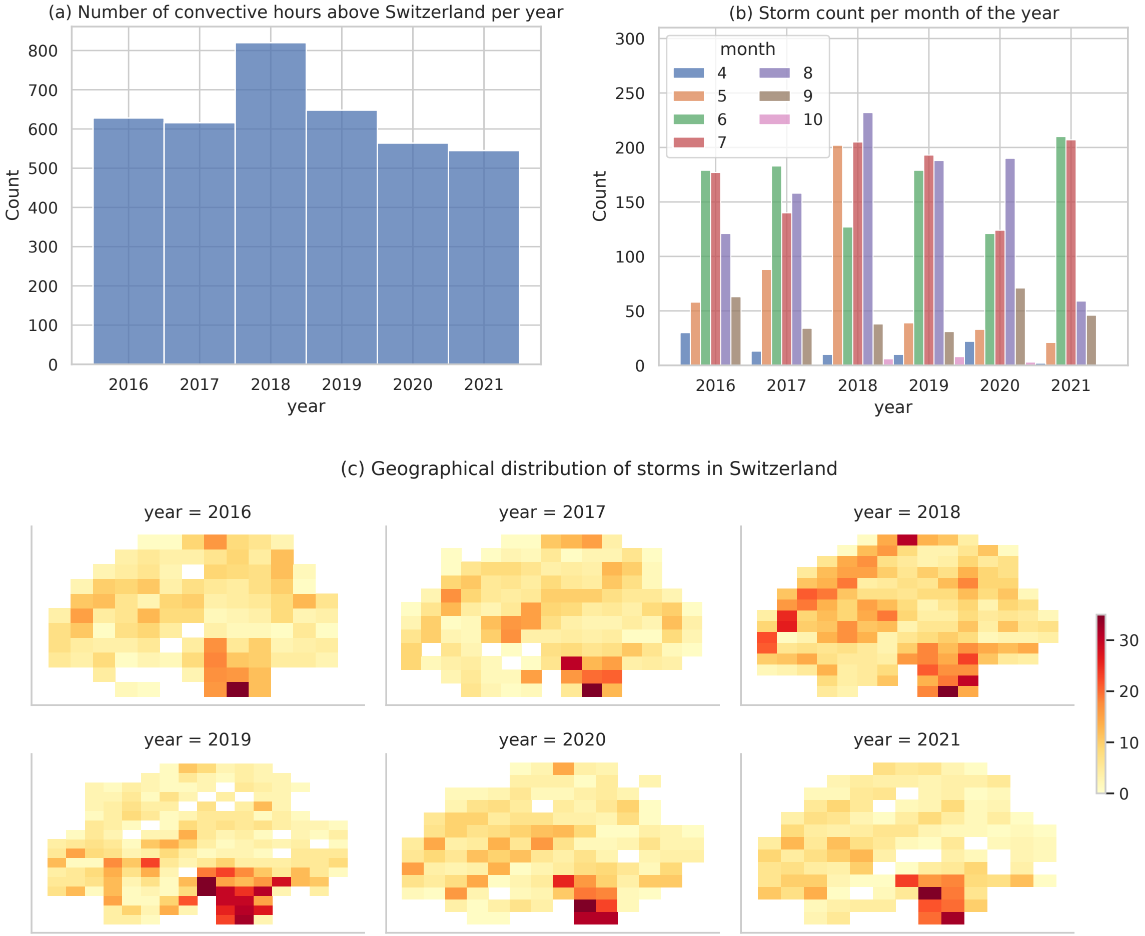

Supplementary Figure 8: 2021 is comparable to years 2016–2020 in terms of number of hours (a), distribution by month (b), and location (c) of convective events.

Second, while 2021 was average in frequency, it nonetheless included several of the most damaging convective events in recent decades, such as the June 28 and July 8 hailstorms [3].

These high-impact events stress test the model's performance.

Third, to mitigate sampling variability, we trained all models using 5-fold cross-validation over the 2016–2020 period. Figures 2 and 3 already report the mean and min/max skill across folds.

Finally, ICON reforecasts and complete SwissMetNet observations were only available for 2021, which constrained our choice of test year for consistency in our benchmarking across models.

References

1. Bi, K. *et al. Pangu-Weather: A 3D High-Resolution Model for Fast and Accurate Global Weather Forecast* en. arXiv:2211.02556 [physics]. Nov. 2022. `http://arxiv.org/abs/2211.02556` (2024).

2. Zhong, X. *et al.* FuXi-2.0: Advancing machine learning weather forecasting model for practical applications. *arXiv preprint arXiv:2409.07188* (2024).

3. Kopp, J. *et al.* The summer 2021 Switzerland hailstorms: weather situation, major impacts and unique observational data. *Weather* **78,** 184–191. eprint: `https://rmets.onlinelibrary.wiley.com/doi/pdf/10.1002/wea.4306`. `https://rmets.onlinelibrary.wiley.com/doi/abs/10.1002/wea.4306` (2023).
